# Exploring Kinetics of Phenol Biodegradation by *Cupriavidus taiwanesis* 187

**DOI:** 10.3390/ijms11125065

**Published:** 2010-12-07

**Authors:** Yu-Hong Wei, Wei-Chuan Chen, Shan-Ming Chang, Bor-Yann Chen

**Affiliations:** 1 Graduate School of Biotechnology and Bioengineering, Yuan Ze University, Chung-Li, Taoyuan 320, Taiwan; E-Mails: itispay@gmail.com (W.-C.C.); s955806@mail.yzu.edu.tw (S.-M.C.); 2 Department of Chemical and Materials Engineering, National I-Lan University, I-Lan city 260, Taiwan

**Keywords:** phenol degradation, biodegradation, Cupriavidus taiwanesis, Haldane’s model, kinetic model, stability analysis

## Abstract

Phenol biodegradation in batch systems using *Cupriavidus taiwanesis* 187 has been experimentally studied. To determine the various parameters of a kinetic model, combinations of rearranged equations have been evaluated using inverse polynomial techniques for parameter estimation. The correlations between lag phase and phase concentration suggest that considering phenol inhibition in kinetic analysis is helpful for characterizing phenol degradation. This study proposes a novel method to determine multiplicity of steady states in continuous stirred tank reactors (CSTRs) in order to identify the most appropriate kinetics to characterize the dynamics of phenol biodegradation.

## 1. Introduction

Phenol and associated phenolic compounds are common constituents of aqueous effluents derived from various industrial processes such as polymeric resin production, petroleum refining, coal gasification, coking, and the manufacture of pharmaceuticals, explosives, plastic and varnish [1,2]. Phenol (or carbolic acid; C_6_H_5_OH), which is both water soluble and highly flammable, is produced on a large scale as a precursor to many compounds. Phenol has a vapor pressure of 0.41 mm Hg and a log octanol/water partition coefficient (Log K_ow_) of 1.46. Phenol and its vapors are corrosive and are rapidly absorbed from the lungs. Orally ingested phenol is highly toxic to humans. Ingestion of 1 g is reportedly lethal, and smaller quantities can still cause symptoms such as muscle weakness and tremors, loss of coordination, paralysis, convulsions, coma, and respiratory arrest [3]. However, due to its low volatility, the inhalation hazard should be limited. Wastewater contains phenol in the range of 5–500 mg/L [4].

Wastewater containing phenol can be treated by adsorption, stripping, chemical oxidation, solvent extraction and biotreatment [5,6]. Although physico-chemical processes are highly efficient, one disadvantage is dilution by some physical processes, and another is formation of toxic intermediates by chemical oxidation [5]. Biodegradation was initially considered the most cost-effective solution to these problems. However, in phenol concentrations of *ca.* 5–500 mg/L, many wild-type microbes use phenol as a carbon and energy source for cell propagation in soil and water [7]. Phenol degradation begins when microorganisms express monooxygenase activity to hydroxylate phenol to form catechol [8]; catechol is then cleaved via dioxygenase oxidation to form *cis*-*cis*-muconic acid or 2-hydroxymuconate semialdehyde (2-HMS) [9,10].

Evidently, the kinetics of phenol biodegradation is inevitably crucial for efficient removal of phenol. That is, understanding the kinetics of cell growth and biodegradation of phenolic compounds is essential for system optimization. Specifically, a good experimental design and careful mathematical interpretation of data are helpful for understanding the dynamic characteristics of phenol degradation when using *Cupriavidus taiwanensis* [10]. Additionally, the rhizobial bacterium *C. taiwanensis* is reportedly efficient in degrading phenol and trichloroethylene (TCE) [10]; it is particularly appropriate for *in situ* or on-site bioremediation of contaminated soil as its nodulation characteristics and nitrogen fixation with its host plants may enhance biodegradation of soil pollutants [10]. Here, the influence of phenol on biodegradation was quantitatively described by several kinetic models (e.g., the Halden model and the Yano model). This study is the first to perform stability analysis of a chemical reactor [11] in order to identify the appropriate kinetics for phenol biodegradation.

## 2. Model description

### 2.1. Microbial Growth

The specific growth rate of cells in a batch system, μ (h^−1^), is defined as [2]

(1) $$\mu =\frac{1}{X}\frac{\text{dX}}{\text{dt}}=\frac{d\text{ln}X}{\text{dt}}$$

where *X* is the cell concentration (g/L). The value of μ is determined at the exponential phase of the growth curve. The change in substrate concentration is defined by

(2) $$\frac{\text{dS}}{\text{dt}}=-\mu \frac{\text{dX}}{Y_{X/S}}$$

where *S* is substrate concentration (mg/L), and *Y_x/s_* is cell yield (g cell/g substrate). The relationship between cell mass formation and substrate consumption can be determined by

(3) $$Y_{X/S}=-\frac{\text{dX}}{\text{dS}}$$

Although *Y_x/s_* is constant, Pirt proposed the following model to determine substrate utilization for cell maintenance [9,12]:

(4) $$\frac{1}{Y_{X/S}}=\frac{1}{Y_{G}}+\frac{m}{\mu }$$

where *Y_G_* is the theoretical cell yield (g cell/g substrate), indicating the maximal conversion of unit substrate to cell mass, and *m* is the specific maintenance coefficient (g substrate/g cell-h). If cell maintenance is not considered (*i.e*., m = 0), cell yield (*Y_x/s_*) is equal to the theoretical cell yield (*Y_G_*).

### 2.2. Substrate Inhibition Model

The model most commonly used to describe the dependence of specific growth rate (μ) on the concentration of an inhibitory substrate (*S*) is the Haldane model [13,14]:

(5) $$\mu =\frac{\mu_{\text{max}}S}{Ks+S+S^{2}/K_{I}}$$

where μ_max_ is the maximum growth rate (h^−1^), *Ks* is the substrate-affinity constant (mg/L), and *K_I_* is the substrate-inhibition constant (mg/L). Several modified models with two or more parameters have also been developed. Other models of enzyme kinetics or alcohol fermentation with inhibitory substrate(s) or product(s) may also be applicable for phenol degradation. Three alternatives (Table 1) were considered in this study.

## 3. Results and Discussions

### 3.1. Cell Growth and Phenol Degradation in Batch Culture

**Figure 1.**
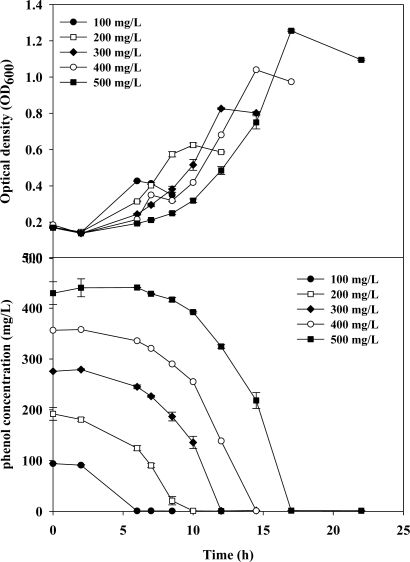
Batch culture and phenol degradation of *C. taiwanesis* 187 on phenol (100–500 mg/L) in flasks (n = 3).

As indicated in Figure 1, in time course experiments investigating cell growth and phenol degradation, *C. taiwanesis* 187 degraded phenol to very low concentrations. When the phenol substrate was depleted, the bacterial cells gradually grew to stationary phase. It was observed that the lag phase was extended when the initial phenol concentration was higher due to the slower cell adaptation. This indicates that the acute toxicity of phenol inhibited *C. taiwanesis* 187 at high concentrations [15].

### 3.2. Evaluation of Kinetic Parameters

**Figure 2.**
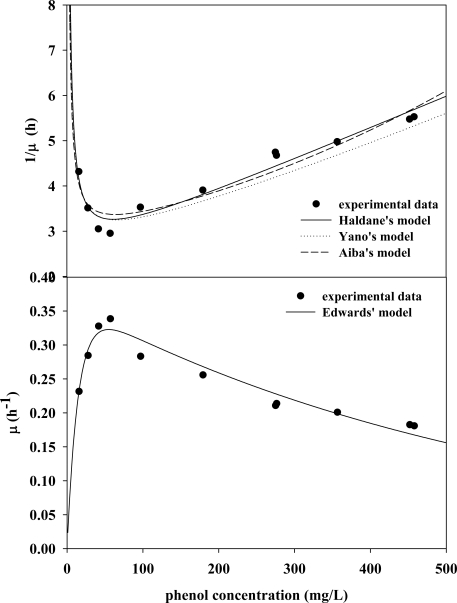
Analysis of the kinetic parameters of different models.

**Figure 3.**
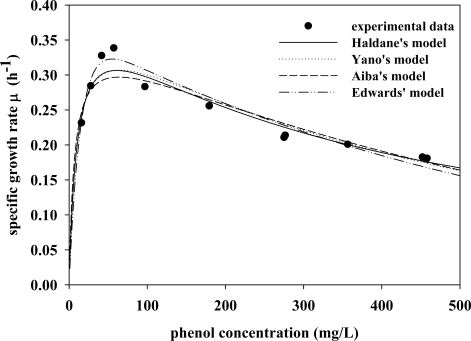
Specific growth rates of *C. taiwanesis* 187 on different initial phenol concentrations with fitting of various kinetic models (n = 3). The model parameters are given in Table 3.

Kinetic parameters were determined using a series of batch cultures at various initial phenol concentrations. The specific growth rates were calculated for different initial phenol concentrations according to the slopes of time-series plots of ln*X* at the exponential growth phase. However, this approach proved unsatisfactory for estimating μ_max_, *Ks*, *K_I_* or *K*, as asymptotes could not be extrapolated accurately at high phenol concentrations. Thus, the validity of the kinetic model could not be confirmed. Inverse polynomials for linear regression technique were therefore applied to the kinetic model (Table 2) so that each asymptote between the experimental data and model prediction could be evaluated via a curve fitting method in MATLAB 6.5 (Figure 2; Table 3). As indicated in Figure 3 and Table 3, specific growth rates could be expressed in terms of the effects of different phenol concentration and the best fit to kinetic parameters. These findings indicated that all kinetic models seemed to be suitable to depict the characteristics of phenol biodegradation and accurately characterized phenol biodegradation. The next question was which kinetic model(s) were mathematically feasible and dynamically viable for characterizing phenol degradation in industrial reactor operations. The Haldane model is frequently cited in the literature due to its mathematical simplicity and wide applicability [2]. The underlying mechanism of the Haldane model can be expressed as follows:

(9a) X+S↔XS→X+P

(9b) $$\text{XS}+S↔{\text{XS}}_{2}$$

where X, S, and P denote *C. taiwanensis* cell, phenol substrate and degraded product (e.g., 2-HMS), respectively. Verifying the validity of this mechanism requires further biochemistry and cell biology studies. However, stability analysis [11] is still applicable for validating which kinetic model is mathematically viable (Figures 2 and 3) and dynamically appropriate for predicting characteristics during practical operation.

### 3.3. Verifying Feasible Kinetic Models

According to Appendix A and Appendix B, the uniqueness condition of steady states (*i.e*., inequality (A-3d)) in CSTR operation [11] for phenol biodegradation under different kinetic conditions can be formulated as follows:

(10a) $$\text{Haldane}\;\text{model}:\;(S-S_{0})\cdot (K_{S}-S^{2}/K_{I})-S(K_{S}+S+S^{2}/K_{I})<0$$

(10b) $$\text{Aiba}\;\text{et}\;\text{al}.\;\text{model}:\;(S-S_{0})\cdot (K_{I}K_{S}-K_{S}S-S^{2})-K_{I}S(K_{S}+S)<0$$

(10c) $$\text{Edwards}\;\text{model}:\;(S-S_{0})\cdot \left(\frac{e^{-S/K_{S}}}{K_{S}}-\frac{e^{-S/K_{I}}}{K_{I}}\right)-(e^{-S/K_{S}}-e^{-S/K_{I}})<0$$

(10d) $$\text{Yano}\;\text{et}\;\text{al}.\;\text{model}:\;(S-S_{0})\cdot \left(K_{S}-\frac{S^{2}}{K_{I}}-\frac{2S^{3}}{K_{I}K}\right)-S\left(K_{S}+S+\frac{S^{2}}{K_{I}}+\frac{S^{3}}{K_{I}K}\right)<0$$

If the inlet phenol concentration is S_0_ = 1000 mg/L, the uniqueness conditions of the steady state (SS) can be obtained through root searching for all equations (unit for S: mg L^−1^) as follows:

Haldane model: 0<S<87.515 or 310.245<S<1000,

Aiba et al. model: 0<S<124.836 or 336.377<S<1000,

Edwards model: 0<S<69.584 or 408<S<1000,

Yano et al. model: 0<S<101.432 or 354.802<S<1000.

To verify the existence of multiple steady states, our earlier study proposed using the experimental technique of dilution shift-up and shift-down [16] in continuous cultures for phenol degradation. If multiplicity of SSs was observed in only one kinetic model and not in others, this kinetic model would clearly be suitable for characterizing phenol biodegradation. As phenol biodegradation is growth-associated [17], the CSTR mode of operation with dilution shift-up and shift-down was used to determine which kinetic model is more appropriate for analyzing phenol biodegradation. These experimental results will be discussed in follow-up studies. For example, if the multiplicity of steady states in CSTR using the technique of dilution shift-up/down would not take place near the perturbation of steady state phenol concentration of *ca.* 120 mg/L, Aiba *et al.*’s model would be the best kinetic model for phenol degradation by *C. taiwanensis* 187.

## 4. Experimental Section

### 4.1. Bacterial Strain and Culture Medium

Two types of media were used to grow indigenous phenol degrader *Caprividus taiwanesis* 187. Luria-Bertani Broth medium (LB) consisting of 5 g/L yeast extract, 10 g/L tryptone and 10 g/L NaCl was purchased from Sigma. The other medium was mineral salt containing various concentrations of phenol (25–500 mg/L) combined with 1.56 g/L (NH_4_)_2_SO_4_, 3 g/L KH_2_PO_4_, 7 g/L Na_2_HPO_4_, 0.011 g/L CaCl_2_·2H_2_O, and 0.246 g/L MgSO_4_·7H_2_O.

### 4.2. Culture Conditions

For cell activation, an appropriate amount of frozen *C. taiwanesis* 187 culture was transferred to LB medium agar and incubated overnight at 30 °C. A single colony of *C. taiwanesis* 187 was then transferred to 3mL LB medium for overnight incubation at 30 °C, 200 rpm. For preculture, 1.0 mL of *C. taiwanesis* 187 was transferred to 50 mL LB flask cultures (*ca*. pH 7.0) and then incubated at 30 °C, 200 rpm for *ca.* 12 h. After preculture, 2 mL of the precultured broth was inoculated into 100 mL mineral salt medium for batch flask cultures under similar culture conditions. During fermentation, samples were taken at designated time intervals to measure cell and phenol concentrations.

### 4.3. Analytical Methods

Cell concentration was measured within the linear range of absorbance (*ca.* 0.1–0.7) using a VIS-UV spectrophotometer (Milton Roy Spectronic 601, Ivyland, PA, U.S.) to determine the optical density at 600 nm (OD_600_). The relationship between dry cell weight (*X*) and OD_600_ of the culture was 1.0 OD_600_ ≅ 0.2853 g DCW/L. The phenol concentration was determined via reverse-phase high performance liquid chromatography (HPLC) (LC-10AT, Shimadzu, Tokyo, Japan) equipped with a Merck C18 column (5 μm, Merck, Germany). The samples were acidified by equivalent volume 2N H_2_SO_4_ and then passed through a 0.2 μm filter. Phenol concentration was analyzed under a linear elution gradient using a solution of methanol, acetic acid and water in a ratio of 50:50:1 (%, v/v). An aliquot of 10 μL of the sample was injected and analyzed by HPLC, and the wavelength for phenol was set to 280 nm as described in [2].

### 4.4. Data Analysis

The specific growth rate (*μ*) was determined during the exponential growth phase by plotting a graph between ln*X versus* time where the slope μ was approximately constant. The initial substrate concentration (*S*_0_) was determined at the beginning of exponential growth phase [4]. The kinetic parameters (e.g., *μ*_max_, *Ks*, *K_I_* and *K*) were determined using curve fitting method in MATLAB 6.5. Cell growth and phenol degradation were simulated using software “ode23” of MATLAB function. The state equations of *X* and *S* was as follows:

In the Haldane model, the equations were reformulated as

(11) $$\frac{\text{dX}}{\text{dt}}=\frac{\mu_{\text{max}}\text{SX}}{K_{S}+S+S^{2}/K_{I}}$$

(12) $$-\frac{\text{dS}}{\text{dt}}=\frac{1}{Y_{X/S}}\frac{\mu_{\text{max}}\text{SX}}{K_{S}+S+S^{2}/K_{I}}$$

For the other kinetic models, the above equations were rewritten as

(13) $$\frac{\text{dX}}{\text{dt}}=\mu X$$

(14) $$-\frac{\text{dS}}{\text{dt}}=\frac{1}{Y_{X/S}}\mu X$$

## 5. Conclusion

The growth kinetics of *C. taiwanesis* 187 during the biodegradation of phenol and the resulting inhibitory effect on cell growth were studied. The duration of the lag phase correlated with phenol concentration. Kinetic parameters of various models were determined using inverse polynomial technique (*i.e.*, Lineaweaver-Burk plot). Time course data were used to compare kinetic models of phenol biodegradation at varying initial phenol concentrations (100–500 mg/L) in batch cultures. We proposed a novel method of identifying kinetics that is most appropriate for characterizing phenol biodegradation in CSTRs.

## Figures and Tables

**Table 1.** Examples of kinetic models for substrate-inhibition.

**Source**	**Model**	**Reference**
Yano *et al.*	(6) $\mu =\frac{\mu_{\text{max}}S}{S+K_{S}+(S^{2}/K_{I})[1+(S/K)]}$	[14]
Aiba *et al.*	(7) $\mu =\frac{\mu_{\text{max}}S}{S+K_{S}}\text{exp}\left(\frac{-S}{K_{I}}\right)$	[14]
Edwards	(8) $\mu =\mu_{\text{max}}\left[\text{exp}\left(\frac{-S}{K_{I}}\right)-\text{exp}\left(\frac{-S}{K_{S}}\right)\right]$	[15]

**Table 2.** The refomulated equation of various models for determining kinetics via the technique of inverse polynomials or non-linear searching.

**Model**	**Rearranged equation**
Haldane model	$\frac{1}{\mu }=\frac{K_{S}}{\mu_{\text{max}}}\left(\frac{1}{S}\right)+\frac{1}{\mu_{\text{max}}K_{I}}S+\frac{1}{\mu_{\text{max}}}$
Yano’s model	$\frac{1}{\mu }=\frac{K_{S}}{\mu_{\text{max}}}\left(\frac{1}{S}\right)+\frac{1}{\mu_{\text{max}}K_{I}}S+\frac{1}{\mu_{\text{max}}K_{I}K}S^{2}+\frac{1}{\mu_{\text{max}}}$
Aiba’s model	$\frac{1}{\mu }=\frac{K_{S}}{\mu_{\text{max}}}\left(\frac{1}{S}\right)\text{exp}\left(\frac{S}{K_{I}}\right)+\frac{1}{\mu_{\text{max}}}\text{exp}\left(\frac{S}{K_{I}}\right)$
Edwards model	$\mu =\mu_{\text{max}}\left[\text{exp}\left(\frac{-S}{K_{I}}\right)-\text{exp}\left(\frac{-S}{K_{S}}\right)\right]$

**Table 3.** Parameter estimates and regression statistics for various substrate-inhibition models.

**Model**	**Estimates**				**r^2^**
	***μ***_**max**_**(h^−1^)**	***K***_***S***_**(mg/L)**	***K***_***I***_**(mg/L)**	***K*(mg/L)**	
Haldane’s model	0.4160	10.87	341		0.9249
Yano’s model	0.3972	9.70	467	1561.47	0.9237
Aiba’s model	0.3640	6.99	638		0.9028
Edwards’ model	0.3630	14.41	592		0.9527

## Nomenclature

*K*: substrate-inhibition constant (mg/L)
*K_S_*: substrate-affinity constant (mg/L)
*K_I_*: substrate-inhibition constant (mg/L)
*m*: specific maintenance coefficient (g substrate/g cell-h)
*S*: substrate concentration (mg/L)
*S_0_*: initial substrate concentration (mg/L)
*t*: time (h)
*X*: cell concentration (g/L)
*Y_XIS_*: cell yield (g cell/ g substrate)
*μ*: specific growth rate (h^−1^)
*μ*_max_: maximum specific growth rate (h^−1^)

## Appendix A: Rolle Theorem ensures uniqueness condition(s) for phenol degradation

A prerequisite theorem for verifying kinetic models should first be defined to determine the uniqueness conditions of steady state(s) in CSTR as follows: Let *F* be continuous on the closed interval [*a*, *b*] and differentiable from (*a*, *b*). If *F*(*a*) = *F*(*b*), then there exists at least one number *c* ∈ (*a*, *b*) such that *F’*(*c*) = 0. That is, if there exists no number *c* ∈ (*a*, *b*) such that *F’*(*c*) = 0, then the relationship *F*(*a*) = *F*(*b*) is simply nonexistent (*i.e.*, steady state multiplicity is not present).

Consider an isothermal CSTR described by the following equation (A-1) for phenol biodegradation. Since the concept of a balance only requires that the time derivative be zero, any solution to

(A-1) $$0=F(S)=q(S_{0}-S)-V\cdot R(S)$$

= (substrate inlet) − (substrate output) − (substrate consumption)

is a steady state. As the reaction-rate equation R(S) is nonlinear in the above substrate concentration, multiple solutions are mathematically possible. Using this criterion, one can determine which reaction rate equation (*i.e*., kinetic model) is superior for characterizing phenol biodegradation as shown.

## Appendix B: Sufficient conditions to guarantee the uniqueness condition(s) of solutions

If *F*(*S*) can be equal to zero (*i.e*., steady state zero) at ≥2 different values of *S* (denoted *S*_1_, *S*_2_), it has a zero derivative somewhere between the values of *S* (*i.e*., *F’*(*C*) = 0 at *C* ∈ (*S*_1_, *S*_2_)). Restated, any criteria that made it impossible for $\frac{\text{dF}(S)}{\text{dS}}=0$ is sufficient to ensure a unique solution to equation.

(A-1’) $$F(S)=V\frac{\text{dS}}{\text{dt}}=q(S_{0}-S)-V\cdot R(S)$$

Consider the derivative of F(S):

(A-2) $$\frac{\text{dF}(S)}{\text{dt}}=-q-V\cdot \frac{\text{dR}}{\text{dS}}$$

and the left-handed side of equation (A-2) can never be zero if either

(A-3a) $$\frac{\text{dR}}{\text{dt}}\geq 0$$

or

(A-3b) $$\frac{\text{dF}(S)}{\text{dt}}=-\left(q+V\frac{\text{dR}}{\text{dS}}\right)<0$$

That is, the inequality (A-3b) can be rewritten as

(A-4) $$\frac{\text{dR}}{\text{dt}}>-\frac{q}{V}$$

The inequality (A-4) can also be combined with equation (A-1) to give

(A-3c) $$\frac{\left(S-S_{0}\right)}{R}\frac{\text{dR}}{\text{dS}}=(S-S_{0})\cdot \frac{d\;\text{ln}\;R}{\text{dS}}<1$$

For phenol biodegradation, the reaction rate can be formulated as

(A-5) $$R(S)=\frac{\mu (S)\cdot X}{Y_{X/S}}$$

If cell concentration *X* and cell yield *Y_X/S_* operated around steady state(s) are insensitive to substrate concentration in kinetic models (e.g., Table 1), Eq. (A-3c) can be reformulated as

(A-3d) $$(S-S_{0})\frac{d\;\text{ln}\mu }{\text{dS}}<1$$

The inequality (A-3d) for uniqueness condition of steady state(s) can be used to verify which kinetic model (e.g., Haldane model or Edward’s model) is best for characterizing phenol biodegradation.
